# 160-fold acceleration of the Smith-Waterman algorithm using a field programmable gate array (FPGA)

**DOI:** 10.1186/1471-2105-8-185

**Published:** 2007-06-07

**Authors:** Isaac TS Li, Warren Shum, Kevin Truong

**Affiliations:** 1 Institute of Biomaterials and Biomedical Engineering, University of Toronto, 164 College Street, Toronto, Ontario, M5S 3G9, Canada; 2 Edward S. Rogers Sr. Department of Electrical and Computer Engineering, University of Toronto, 10 King's College Circle, Toronto, Ontario, M5S 3G4, Canada

## Abstract

**Background:**

To infer homology and subsequently gene function, the Smith-Waterman (SW) algorithm is used to find the optimal local alignment between two sequences. When searching sequence databases that may contain hundreds of millions of sequences, this algorithm becomes computationally expensive.

**Results:**

In this paper, we focused on accelerating the Smith-Waterman algorithm by using FPGA-based hardware that implemented a module for computing the score of a single cell of the SW matrix. Then using a grid of this module, the entire SW matrix was computed at the speed of field propagation through the FPGA circuit. These modifications dramatically accelerated the algorithm's computation time by up to 160 folds compared to a pure software implementation running on the same FPGA with an Altera Nios II softprocessor.

**Conclusion:**

This design of FPGA accelerated hardware offers a new promising direction to seeking computation improvement of genomic database searching.

## Background

The Smith-Waterman (SW) algorithm is a well-known algorithm in bioinformatics that finds the optimal alignment between two DNA or protein sequences (the target sequence and the search sequence) [1]. Determining how well two sequences align is important in discovering homologous genes and studying the evolutionary history of molecules and species [2]. However, the SW algorithm is not commonly used to search sequence databases because it is too slow when executed against many sequences. Instead, faster heuristic algorithms such as FASTA [3] and BLAST [4] are used, even though they can not guarantee that the score for the optimal local alignment will be found. Therefore, to achieve both increased speed and the optimal alignment score, it is necessary to develop an approach to reduce the processing time of the SW algorithm. The SW algorithm first creates a two-dimensional (2D) matrix with size equal to the lengths of the two DNA sequences. The score of each cell in the matrix is calculated from neighbouring cells. The optimal alignment score between the two DNA sequences is the highest score in the matrix and the corresponding alignment is determined by back-tracing from the cell with the highest score to the first cell with a zero score.

Many attempts have been made to accelerate the SW algorithm using either software or hardware by focusing on parallel processing of the score matrix [5]. This has been implemented using VLSI (Very Large Scale Integration) [6] and FPGA (Field Programmable Gate Array) [7] by simultaneously evaluating the cells along the minor diagonal of the score matrix. Alternative implementations have been used recently to accelerate the SW algorithm using software parallel programming on common microprocessors with a speed improvement up to six-fold [8]. Here, we dramatically reduced the computation time of the SW algorithm using an FPGA. Our implementation uses custom instructions to accelerate cell scoring in the SW matrix and divides the SW matrix into grids of 8 by 8 cells. Our approach is different from previous FPGA approaches in that the cell scores in each grid are calculated through unclocked signal propagation within the FPGA circuit, whereas previous methods process the minor diagonal values synchronously by the clock. Using our approach, we reduced the expensive writing and reading time of intermediary data between each computation of the diagonals. Furthermore, we eliminate the overestimation of the computation time of the circuit caused by a clock. The cost of this improvement is utilizing more logic elements on the FPGA.

## Results

### Smith-Waterman algorithm

**Figure 1:**
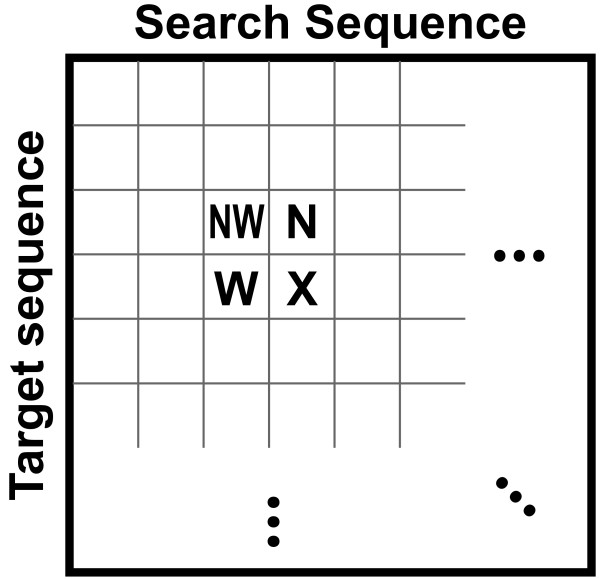
**Basic structure of the SW matrix**. Each cell records the score of the SW matrix, which depends on the search and target sequences. NW, N, and W are cells to the northwest, north, and west of the cell of interest X.

The SW algorithm belongs to a class of algorithms known as dynamic programming. Dynamic programming is used when a large search space can be structured into a succession of stages such that the initial stage contains trivial solutions to subproblems [9]. Typically, this involves structuring the problem to an iterative calculation of cells in a scoring matrix. The following is the commonly used scheme to compute the score of a single cell, score_x, in the score matrix:

score_x = max {score_nw + match_bonus,

   score_nw + mismatch_penalty,

   score_n - opening_gap_penalty - extension_gap_penality,

   score_w - opening_gap_penalty - extension_gap_penality,}

score_nw, score_n and score_w are the scores of the cells to the upper-left (NW), above (N) and left (W) of cell X, respectively (Figure 1). For simplicity, in our case, the match_bonus was 1 if the additional letters to the alignment are equal; the mismatch penalty was 1 if letters are not equal; the opening_gap_penalty was 1; the extension_gap_penalty was 0.1 for each additional gap. Thus, the score of each cell in the 2D matrix (except for the upper left corner) is calculated by three of its neighbouring cells.

### Software implementation

A pure software implementation of the SW algorithm was developed in the C language to benchmark against FPGA-based implementations. A single_cell_module (SCM) was programmed containing the following I/O parameters: score_nw, score_n, score_w, flag_nw, flag_n, flag_w, flag_gap and result_score. The input parameters score_nw, score_n and score_w are scores of the NW, N and W neighboring cells, respectively. The input parameters flag_nw, flag_n, and flag_w indicate the direction of the gap (00_2 _if no gap; 01_2_, gap from the target sequence; 102, gap from the search sequence) of the NW, N and W neighboring cells, respectively. Since the direction of the gap is known from the neighboring cells by the flags, we can determine if the incremental gap penalty of the cell of interest is an opening or extension gap penalty.

Thus, we can perform an affine gap penalty. The output parameters (score_gap and result_score) give the direction of the gap and the final score of the cell of interest, respectively.

The program first loads the target and search sequences into local memory from two text files stored in the flash memory. Then, their sequence lengths are determined and the scoring and gap matrices are created with dimensions of the above sequence lengths. Next, the score of each cell in the SW scoring matrix is calculated using the SCM. Lastly, the completed SW scoring matrix outputs the highest score in the matrix.

### Custom instruction (CI) for SCM using an FPGA

Since calculating the SCM in the SW scoring matrix is repeated, this routine is a good candidate for FPGA-based hardware acceleration. The reconfigurable logic elements in FPGA can be optimally configured to run specific functions through the implementation of custom microprocessor instructions, which are assigned logic elements that perform user-defined operations. Custom instructions, in particular, allow the passing of multiple inputs and outputs in a single clock cycle while the pure software implementation using a conventional microprocessor is limited by the instruction set.

**Figure 2:**
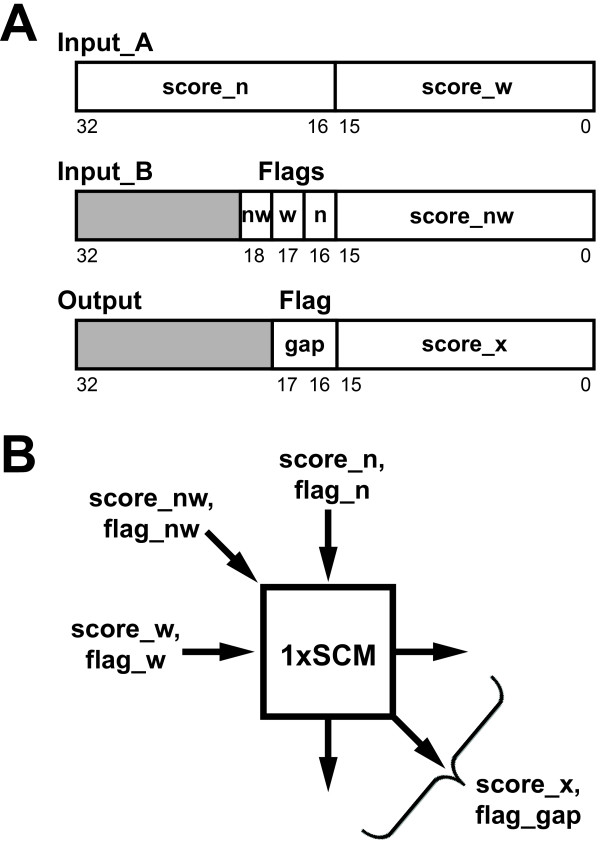
**1×SCM I/O instruction and arrangement**. A. Bit partition of custom instruction for the 1×SCM. The input_A and input_B are two 32-bit data containing the scores and flags from the three neighbouring cells (north, northwest and west). The output is one 32-bit data containing the final scores and the direction of alignment gap. The grey areas indicate the unused data bits. B. Schematic design of the inputs and outputs from one 1×SCM.

The SCM in the pure software implementation was converted to an equivalent FPGA-based custom instruction (hereafter, called 1×SCM) written in the Verilog hardware description language. Since the format for the custom instruction provided by our FPGA board (Altera Stratix) only permits two 32-bit inputs (Input_A and Input B, Figure 2) and our 1×SCM requires 6 inputs (3 scores and 3 flags), the inputs are partitioned and rearranged to be all read in a single clock cycle. Recall that the inputs for the SCM in the pure software implementation are score_nw, score_n, score_w, flag_nw, flag_n, and flag_w. Using bit masking and shifting bit operations, all input scores and their flags are passed to the 1×SCM of cell of interest in one clock cycle (Figure 2). The CI produces the cell score and flags quickly because it makes use of custom hardware, rather than using the standard instruction set of the Nios II as in the software version. The maximum field propagation delay of the 1×SCM was estimated to be 21 ns. Thus, the clock speed of this computation could be no faster than 47.6 MHz. Using the 1×SCM, we computed the cell score and gap flag calculations using a single instruction rather than several.

Lastly, we added the CI for the scoring of a single cell to the instruction set of the Nios II soft microprocessor on the FPGA, so that it can be called in a C program. The flow of computation is identical to that of the software implementation, except that instead of calling a function which describes the SCM, we call the CI.

### A grid design of SCMs using an FPGA

**Figure 3:**
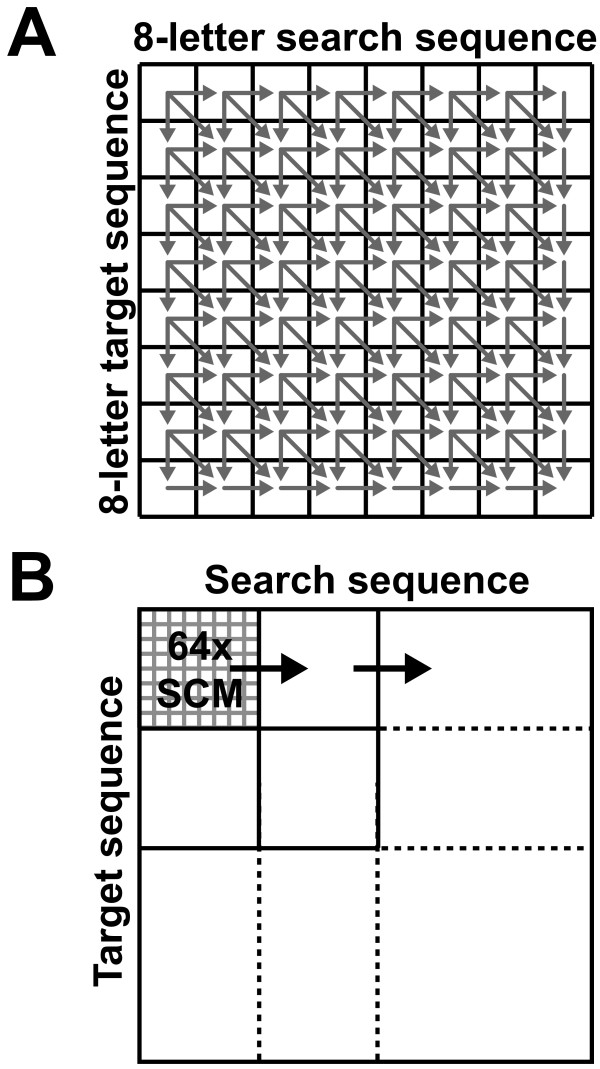
**64×SCM signal and computation propagation**. A. One 64×SCM module aligns an 8-character partial search sequence to an 8-character target sequence. The arrows show the propagation directions of the signals. Because the 64×SCM is unclocked, there is no pre-determined path of propagation. B. When the search and/or target sequences are greater than 8, the scoring matrix is partitioned into many 8 by 8 segments, each to be computed by the 64×SCM.

To further improve the computation speed, we combined 64 instances of 1×SCM into an 8 by 8 grid module (hereafter, called 64×SCM) (Figure 3A), the maximum size allowed by our FPGA board. We programmed the FPGA such that within the grid, the score update of each 1×SCM is not synchronized to a clock, but rather triggered by the changes of scores in neighbouring cells in the W, NW and N direction (Figure 3A). This asynchronous data processing method allows scores to propagate throughout the grid as fast as the field propagation speed allows in the FPGA logic gates, hence drastically improving the computation speed. This implementation can be thought of having all 64 1×SCMs processing at the same time, while the score updating propagates in the grid. The SW matrix is divided into as many grids as needed, which are then calculated with 64×SCM one by one. Because all logic circuits are connected inside the 64×SCM, it takes only one clock cycle to compute the entire 8 by 8 grid. The maximum field propagation delay of the 64×SCM was estimated to be 324 ns. Thus, the clock speed of this computation could be no faster than 3.1 MHz.

**Figure 4:**
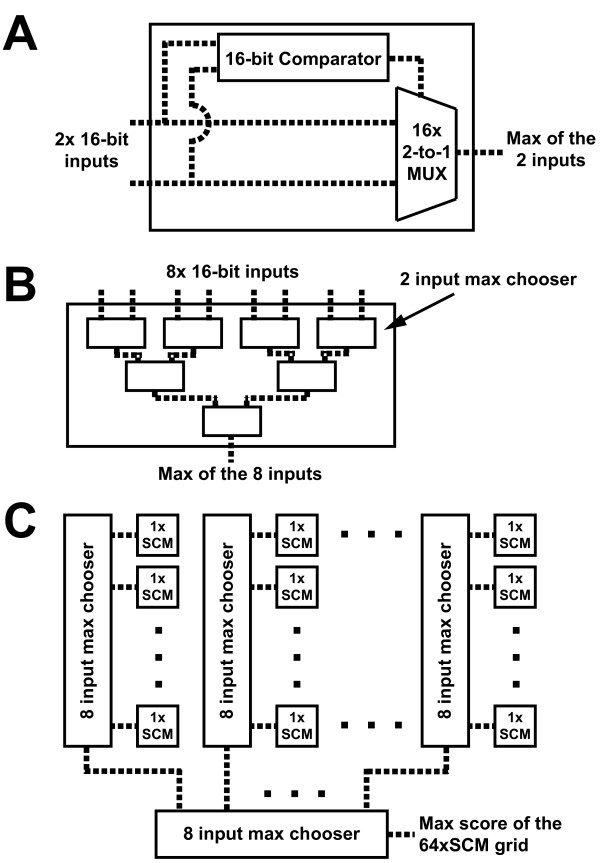
**The max-finder implementations in 64×SCM**. A. The 2-input max-finder circuit implementation. Both inputs and the output are 16-bit data representing the SW matrix score. B. Construction of an 8-input max-finder using 2-input max-finders. C. Max score computation of a 64×SCM. The scores of each column of cells in 64×SCM are inputted to a custom designed 8-input max-finder, the outputs of the 8 columns are then compared against each other using another 8-input max-finder. The output of the last comparator gives the highest score of the 64×SCM.

**Figure 5:**
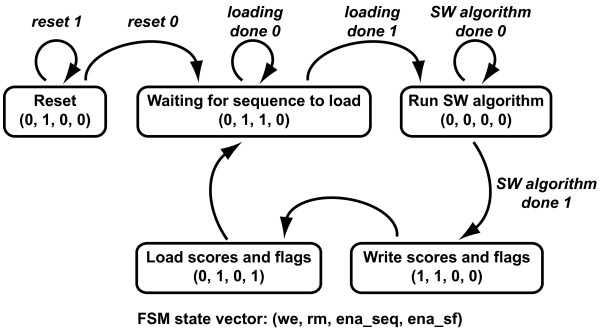
**State diagram of the finite state machine (FSM)**. The states of the moore-type FSM are in the rounded rectangles. The output at each state are defined by the following vector - (we = write enable for SRAM blocks, rm = reset 64×SCM matrix, ena_seq = enable sequences to be loaded, ena_sf = enable scores and flags to be loaded). To clear all scores and flags from the matrix, the FSM is set to the 'Reset' state. Next, the FSM remains in the 'Wait for Sequence Load' state until two sequences of length 8 or less have been loaded by the C program. Once this loading is completed, the C program will assert the done_load signal. At this point, the FSM releases the matrix's reset signal which causes the sequences, scores and flags to propagate through the matrix. After a set delay determined by the critical path of the circuit, the FSM asserts the done_sw signal, and enables the values just calculated to be written into the RAM. Theses scores and flags will be read from the RAM for the next block. The FSM then returns to the 'Wait for Sequence Load' state, and waits for the next length of sequences to come from the C program. This loop is repeated until the entire Smith-Waterman matrix has been calculated and the score of the optimal alignment has been determined. Finally, the results are printed to a command window on the computer. The FSM can be reset by writing to a status register, allowing the matrix to be used for another set of sequences.

As input, this module requires segments of the search and target sequences with a length of up to 8 characters (the length and width of 64×SCM). Also, it requires the scores and gap flags stored from prior 64×SCM calculations in the NW, N and W direction. A second module was created to calculate the maximum score of the 64×SCM by a cascade of max-finders that first finds the maximal score of each column and then finds the maximum of the columns to determine the overall maximum (Figure 4). In order to process sequences longer than the dimension of 64×SCM (in this case, 8), a controller module was programmed to reuse the 64×SCM. This module included a SRAM (static random access memory) block to store scores and gap flags from previous 64×SCM calculations as well as a finite state machine (FSM) to control loading and storing values to the SRAM (Figure 5). Lastly, to access this hardware from a C program, a final interface module defines a set of registers to hold the sequences, lengths, various flags and the final score.

The flow of computation of this hardware controlled from a C program is as follows. First, the search and target sequences are loaded from flash memory and copied to local memory. Once this is done, an on-chip timer is started. Second, score and gap matrices are initiated and the values reset. Third, the search and target sequences are encoded by a custom instruction and loaded into the 64×SCM with their lengths. DNA bases are encoded into two bits (A = 00, T = 01, G = 10, C = 11). Lastly, the result propagates through the grid and completes in a time determined by the field propagation delay. If the sequences are longer than 8 characters, steps 3 and 4 are repeated for the next grid (Figure 3B). Once all grids have finished, the timer is stopped and the running time is displayed on the screen.

### Testing

We tested and compared the performance of the three implementations (pure software, 1×SCM, and 64×SCM) for aligning two DNA sequences with identical lengths ranging from 1 to 1024 base-pairs. We performed the same input for each implementation and measured the time to complete the computations (Table 1). Alignment of the each sequence length was performed three times to produce the statistical variance, which was less than 0.5%. The scoring matrices from the three implementations were compared to ensure identical alignment results. The performance of the implementation was found to be independent to the sequence-similarity between the two DNA sequence queries (data not shown).

**Table 1:** Computation time comparison

**Number of cells**	**Pure software (ms)**	**1×SCM (ms)**	**64×SCM (ms)**
1	0.0244	0.0106	0.0141
4	0.0432	0.0194	0.0135
16	0.120	0.0568	0.0124
64	0.400	0.204	0.0128
256	1.55	0.809	0.0233
1024	6.57	3.45	0.0591
4096	26.2	14. 5	0.193
16384	107	58.3	0.687
262144	1919	1126	11.3
1048576	7719	4504	42.8

**Figure 6:**
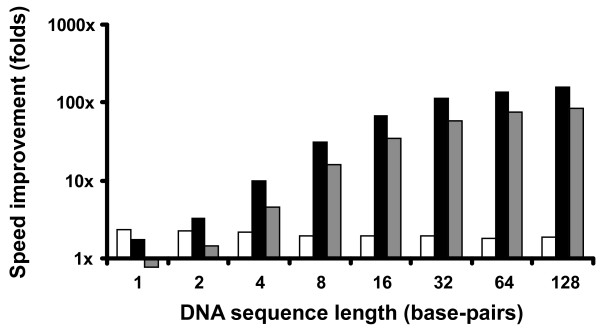
**Speed improvements**. Computation speed improvements (in folds) of the three implementations. Black, 64×SCM over pure software; grey, 64×SCM over 1×SCM; white, 1×SCM over pure software.

**Figure 7:**
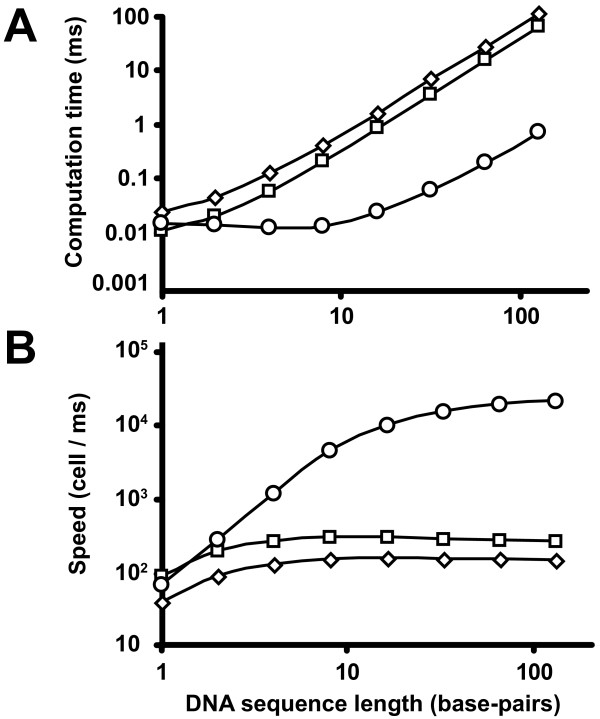
**Computation speed and time**. A, Computation time (in milliseconds) and B, speed (cell evaluated per millisecond) as functions of query DNA sequence length (in base-pairs). Circles are the 64×SCM implementation; squares are 1×SCM implementation; and diamonds are pure software implementation.

The 1×SCM implementation produced a maximal 2-fold speed improvement over the pure software implementation running on the same FPGA with an Altera Nios II softprocessor, while the 64×SCM implementation produced a maximal of 160-fold improvement over the pure software implementation (Figure 6). When the sequence length was smaller or equal to the size of the 64×SCM implementation, the computation time did not increase as the length of the sequence increased (Figure 7A). In comparison to the pure software and 1×SCM implementations, the computational time increased proportionally to square of the sequence length. When the sequence length was larger than the size of the 64×SCM implementation, the slopes of the log (computation time) vs. log (sequence length) graphs of all three implementations approach the same value (Figure 7A) and the speed per cell approaches a constant value (Figure 7B). Thus, in this case, the computation time of all three implementations increased proportionally to square of the sequence length. This is expected as the field propagation of the 64×SCM implementation is restricted to the 8 by 8 grid. However, if we increase the size of the grid to cover the average size of sequences comparisons (for example, one thousand base-pairs), we could significantly improve the computation time for the majority of sequence alignment queries.

## Discussion

While this FPGA implementation using a single Altera Stratix FPGA board does not compute cells per second in a comparable speed to existing software implementations, it is likely that expanding this design to state-of-the-art FPGA architectures will outperform them. Rognes and Seeberg's software implementation running on a Pentium III 500 MHz processor (2000) showed a performance of 150 million cells per second [8], while recently Farrar's software implementation running on a Xeon Core Duo 2 GHz processor (2007) showed a performance of 3,000 million cells per second [10]. In Table 1, 1048576 cells were computed by the 64×SCM implementation in 42.8 ms, which is equivalent to 24.5 million cells per second. It is important to note that most of the processing time is consumed by writing and reading intermediary data to and from static RAM from multiple executions of the 64×SCM module that is necessary when computing a SW matrix that is larger than an 8 by 8 grid size. For example, the computation of a SW matrix with grid size 400 by 400 would require 2500 executions of the 64×SCM module. This loading and reading time could be dramatically reduced by using a larger grid size encompassing the average length of query sequences, perhaps 1000 by 1000 cells. Using this grid size, the computational time is the initial load time of sequence data added with the field propagation delay across the grid which is at most 1000 1×SCM across and 999 down for a total of 1999 1×SCM propagation delays. Our above estimation for the maximum field propagation delay of one 1×SCM on our FPGA board is 21 ns. If that estimate is used, the computation of the 1000 by 1000 grid will be completed in at most 41,979 ns. This corresponds to a computation speed of 23,800 million cells per second. This would actually be an underestimate in what can be achieve in state-of-the-art FPGA boards as the field propagation delay is faster because the density of the transistors is higher and therefore field propagation distance is shorter.

While a 1000 by 1000 grid size is desired, the 8 by 8 grid size was limited by our FPGA board because it only has 40,000 logic elements. A single 1×SCM utilizes 267 logic elements. If only 1×SCM modules were programmed on this FPGA board, there could be a maximum of ~150 1×SCM, however only 64 1×SCMs could be programmed because other internal FPGA hardware requires logic elements as well. One way to reduce logic element utilization is to decrease the bit-size of the score. Currently, state-of-the-art FPGA hardware (such as Starbridge Systems, HC-62) have 11 Xilinx Virtex II FPGAs on single board for a total of 62 million logic elements.

This FPGA hardware could create a maximum of ~230,000 1×SCM. Thus, we could potentially have a maximum grid size of 480 by 480. With ever improving hardware, the gap to a 1000 by 1000 grid size will inevitably be bridged.

## Conclusion

Since the SW algorithm becomes computationally expensive for comparing sequences in a large database, we accelerated the computation time by using FPGA hardware. To quantitatively assess the computational improvement, we compared an implementation of the algorithm in pure software running on the Altera Nios II softprocessor with FPGA-based implementations that had a SCM CI (1×SCM) and a SCM grid (64×SCM). The implementation with a 64×SCM accelerated the algorithm by a maximal of 160-fold in sequence lengths less than or equal to the grid length. The computation is significantly improved because it is occurring as fast as electrons can propagate through the FPGA circuit. By using a grid length that is the size of an average sequence length, the computation time of the average sequence comparison can be further significantly improved. Thus, expanding our FPGA design to more powerful FPGA systems with parallel and higher density logic elements is a promising direction to significantly improve genomic sequence searching.

## Methods

The Altera Stratix EP1S40 FPGA was used for development and evaluation with Verilog was as the hardware description language. Using both the Altera Quartus II software and Nios II Integrated Development Environment (IDE), the Nios II softprocessor was programmed onto the FPGA to execute C programs that use custom FPGA hardware for the acceleration of the algorithm. Altera Quartus II and Nios II software were running on a Dell OptiPlex computer with a 2 GHz Intel Pentium 4 processor, 512 MB RAM, 40 GB hard drive and a Windows XP operating system.

## Availability and requirements

**Name: **FPGA-accelerated Smith-Waterman algorithm

**Web address: **

**Operating system requirement: **Windows XP

**Programming language: **C for software implementation, Visual Basic 6.0 for graphical user interface, Verilog for hardware description language

**Hardware requirements: **At least Altera Stratix FPGA board

**Software requirements: **Altera Quartus II and Nios II IDE

**License: **Open-source and free for academic and non-profit use.

## Authors' contributions

ITSL carried out the hardware characterization and drafted the manuscript. WS carried out the hardware implementation. KT supervised the project development.
